# Echocardiographic changes after arteriovenous fistula creation in hemodialysis patients 

**DOI:** 10.5414/CN110816

**Published:** 2022-09-28

**Authors:** Karen Manzur-Pineda, Laisel Martinez, Roger Alvarez, Adriana Dejman, Roberto I. Vazquez-Padron, Marwan Tabbara, Juan C. Duque

**Affiliations:** 1 DeWitt Daughtry Family Department of Surgery, Division of Vascular and Endovascular Surgery,; 2 Kats Family Division of Pulmonary, Allergy, Critical Care and Sleep Medicine, and; 3 Katz Family Division of Nephrology and Hypertension, Leonard M. Miller School of Medicine, University of Miami, Miami, FL, USA

**Keywords:** pulmonary hypertension, echocardiogram, right ventricular systolic pressure, left ventricular mass, arteriovenous fistula

## Abstract

Background: Pulmonary hypertension (PH) is common in end-stage renal disease (ESRD) patients and is associated with increased all-cause and cardiovascular mortality in this group. There is scarce data on the long-term effect of arteriovenous fistula (AVF) creation on pulmonary hypertension (PH) and the reflected changes in echocardiographic measurements. Materials and methods: This is a retrospective study of 54 patients who underwent AVF creation between 2009 and 2014 and with echocardiographic evaluations before and after surgery. We analyzed pairwise changes in right ventricular systolic pressure (RVSP), right atrial pressure (RAP) during systole, left ventricular mass (LVM), tricuspid regurgitation (TR), mitral E/E’ ratio, and ejection fraction (EF), as well as the factors that predicted change in RVSP after surgery. Results: The median time for the preoperative echocardiogram was 0.3 years (interquartile range (IQR) 0.2 – 0.7 years) prior to AVF creation, while the follow-up echo was done 1.3 (0.6 – 2.1) years after surgery. 67% of the patients had RVSP > 37 mmHg at baseline. There was a significant reduction in RVSP after AVF creation compared to baseline (median 33 (IQR 26 – 43) vs. 46 mmHg, p = 0.0015), with 59% of the patients experiencing a decrease and 19% remaining stable. There were also significant decreases in LVM (201 (143 – 256) vs. 215 (163 – 276), p = 0.045) and RAP systole (10 (10 – 15) vs. 3 (3 – 8); p < 0.001) after surgery. Higher preoperative weight (p = 0.038) and RVSP (p = 0.006), and use of loop diuretics (p = 0.015) were significantly associated with improvement in RVSP after AVF creation. Conclusion: Our results suggest that AVF creation is associated with a significant reduction or stable measurements of RVSP in the ESRD population, likely due to an improvement in volume status.

## Introduction 

Pulmonary hypertension (PH) is a chronic and progressive disease characterized by increased blood pressure in the pulmonary vascular bed that can eventually lead to right heart failure and death [1]. The World Health Organization (WHO) classifies PH into five subgroups based on the main etiology. These include pulmonary arteriopathy (WHO Group 1), left heart disease (Group 2), chronic lung disease and/or hypoxia (Group 3), chronic thromboembolic PH (Group 4), and PH secondary to other causes (Group 5) [2]. The latest group of unclear and/or multifactorial mechanisms includes PH due to chronic kidney disease (CKD) and end-stage renal disease (ESRD), a classification that reflects the complexity of contributing factors in the CKD population. 

Pulmonary hypertension was previously defined as a mean pulmonary arterial pressure (mPAP) ≥ 25 mmHg at rest as assessed by right heart catheterization (RHC) [2, 3]. However, in 2019 the 6^th^ World Symposium on Pulmonary Hypertension Task Force redefined PH as an mPAP > 20 mmHg and additional hemodynamic criteria, such as pulmonary vascular resistance (PVR) and pulmonary arterial wedge pressure (PAWP) depending on its pre- or post-capillary origin [4]. Although RHC is the gold standard for diagnosis, initial evaluation and follow-up with echocardiography in most asymptomatic patients are reasonable [5]. Similar to the recent changes to the RHC-based definition, multiple studies have called for lowering the echocardiogram-based right ventricular systolic pressure (RVSP) threshold for PH from 40 mmHg to 30 – 39 mmHg [6, 7, 8], and at minimum to provide further clinical evaluation > 27 mmHg [6]. 

Pulmonary hypertension is associated with increased cardiovascular and all-cause mortality in ESRD patients [9, 10, 11, 12, 13]. The prevalence of PH ranges from 9 to 39% in advanced CKD and 19 – 77% in hemodialysis patients [11, 12, 13, 14, 15, 16, 17]. Post-capillary PH is more common than pre-capillary etiology [10, 12, 14], although certain patient subpopulations such as women are overrepresented in the latter [10]. It is widely believed that arteriovenous fistula (AVF) creation and long-term hemodialysis contribute to the pathogenesis and/or exacerbate PH [15, 18, 19, 20, 21, 22]. Increased venous return and cardiac output are commonly blamed for this phenomenon [18, 23]. However, associations between AVF flow and pulmonary artery pressure (PAP) have been inconsistent across studies [14, 24, 25, 26, 27, 28], and analyses of other clinical factors suggest that the fistula by itself may not be the primary culprit in PH [15, 29, 30, 31, 32, 33]. 

One of the confounding problems is the scarcity of studies with baseline pulmonary pressure measurements prior to AVF creation [25, 28, 29, 30]. Without this information, we may be overestimating the effect of the fistula. In this retrospective study, we compared pulmonary pressures by echocardiography before and after AVF creation and followed the evolution of RVSP as the main PH parameter with the use of the fistula for hemodialysis. 

## Materials and methods 

### Study participants 

This retrospective study included 93 patients who underwent AVF creation at the University of Miami Hospital or the Jackson Memorial Health System between January 2009 and December 2014. Inclusion criteria consisted of patients 18 years old or older with an echocardiogram performed before and after AVF creation. From these, the statistical analyses included 54 patients with the pre-surgical echocardiogram within 1 year prior to AVF creation and the postoperative follow-up echo up to 3 years after surgery. Demographic and clinical data were collected including patient’s age, ethnicity, comorbidities, AVF anastomosis, access location, and two-dimensional (2D) echocardiogram measurements. The study was approved by the Institutional Review Board at both institutions. 

### Echocardiographic measurements 

Echocardiograms were performed using standard M-mode and 2D images. All measurements were obtained by a trained cardiologist in the echocardiography service as recommended by the American Society of Echocardiography [34]. RVSP values > 37 mmHg were considered positive for PH [35]. Left ventricular mass (LVM) was calculated from 2D-echo measurements using the equation LVM = 0.8 (1.04 [(LVEDD + IVSd + PWd)^3-^LVEDD^3^]) + 0.6, where LVEDD is left ventricular end-diastolic dimension; IVSd, interventricular septal thickness at end-diastole; and PWd, posterior wall thickness at end-diastole [36, 37]. Stroke volume (SV) was calculated from 2D-echo measurements using the equation SV = EDV-ESV, where EDV is end diastolic volume and ESV is end systolic volume [38]. 

### Statistical analyses 

Statistical analyses were performed using STATA (version 17, College Station, TX, USA) and GraphPad Prism 8.4.0 (San Diego, CA, USA). Continuous variables are presented as mean ± standard deviation (SD) when normally distributed or median (interquartile range (IQR)) if not normal. Pre vs. post comparisons of echocardiographic parameters were performed using paired t-tests or paired Wilcoxon signed-rank tests as appropriate. An increase in RVSP after AVF creation was defined as an increment > 3 mmHg compared to the baseline value, given the inter-observer variability range reported by Patton et al. [39] in trained personnel. Similarly, a decrease in RVSP was defined as a reduction by more than 3 mmHg, while post-AVF values within 3 mmHg of baseline measurements were considered “stable”. Statistical associations of baseline clinical variables and change in RVSP (calculated as postoperative value minus baseline RVSP) were assessed using a multivariate general linear regression model adjusting for demographics, comorbidities, vascular access history, AVF features, and baseline RVSP. Statistical associations between other echocardiographic measurements (LVM, stroke volume, RAP systole, mitral E/E’ ratio) and change in RVSP were assessed using a multivariate general linear regression model adjusting for baseline measurements, pre-post change of select parameters (stroke volume and E/E’), medications at the time of access creation, preoperative weight, pre-dialysis status, and baseline RVSP. A p-value < 0.05 was considered significant. 

## Results 

### Baseline characteristics of the study population 

A total of 54 patients had 2D-echocardiograms performed within the year prior to and up to 3 years after AVF creation (Table 1, Table 2). The median time for the pre-surgical echo was –0.3 years (IQR –0.7 to –0.2), while the postoperative follow-up was done 1.3 years after surgery (Table 2). The study cohort had a mean age of 51 ± 13 years, 43% were female, and predominantly African American (57%) or Hispanic (35%). The average body mass index for the study cohort was 27.8 (± 6.4) kg/m^2^. 98% of the patients had a medical history of essential hypertension, 69% were diabetic, 22% had coronary artery disease, and 19% had congestive heart failure. In terms of blood pressure and volume control measurements, 76% of the patients were on β-blockers, 64% on calcium channel blockers, 45% on angiotensin-converting enzyme inhibitors or angiotensin receptor blockers (ACEI/ARB), 40% on loop diuretics, and 40% on vasodilators such as hydralazine (Table 1). 

Over 1/4 of the subjects (26%) had not initiated hemodialysis at the time of AVF creation. The remaining patients were receiving hemodialysis via a tunneled dialysis catheter and/or had a history of a previous AVF (9%). The inflow of the fistula was brachial-based in 82% and radial-based in the rest of the newly created vascular accesses. The location of AVF was most prevalent in the left than the right upper extremity, with 81 and 19%, respectively (Table 1). 

### Echocardiographic changes after AVF creation 

**Figure 1:**
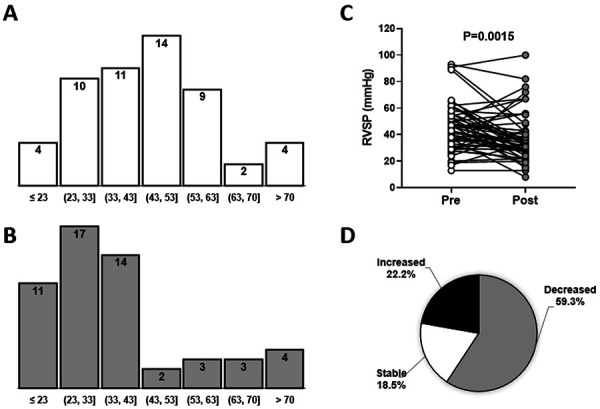
Pre-post change in right ventricular systolic pressure (RVSP) after arteriovenous fistula (AVF) creation. A, B: Histograms of RVSP measurements prior to (A) and after (B) AVF creation in 54 patients. C: Paired comparison of RVSP at two time points using a Wilcoxon matched-pairs signed-rank test. D: Pie graph of patients who experienced an increase in RVSP after AVF creation (defined as increment > 3 mmHg), a decrease (reduction by more than 3 mmHg), or for whom RVSP values remained stable (post-measurement within 3 mmHg of baseline value).

67% of the patients had elevated RVSP (defined as RVSP > 37 mmHg [35]) and 91% had high LVM (LVM > 122 or 149 g for females and males, respectively [36, 37]) at the time of fistula creation (Table 1) (Figure 1A). Pairwise comparisons of echocardiographic parameters demonstrated a significant decrease in RVSP (33 (26 – 43) vs. 46 mmHg, p = 0.0015) after AVF surgery (Table 2) (Figure 1B, C), with 59% of the patients experiencing a decrease and 19% remaining stable (defined as a follow-up value within 3 units of baseline; Figure 1D). In the 12 patients (22%) who experienced an increase in RVSP after surgery (Figure 1D), this increment ranged from 4 to 46 mmHg. 

There were also significant reductions in LVM (215 (163 – 276) vs. 201 (143 – 256) g, p = 0.045) and right atrial pressure (RAP) during systole (10 (10 – 15) vs. 3 (3 – 8) mmHg; p < 0.001) after surgery. A non-significant trend toward lower tricuspid regurgitation (TR) (36 (27 – 42) vs. 29 (23 – 44) mmHg; p = 0.093) was also seen with AVF creation. No significant differences were noted between pre- and post-fractional shortening, TR peak velocity, stroke volume, E/E’ ratio, and ejection fraction (Table 2). 

In 32 patients with a subsequent follow-up echo within 2 years of the first postop echocardiogram (median time 2.6 years (2.1 – 3.5) after AVF creation), the median RVSP was equivalent to the prior echocardiogram (36 (30 – 52) vs. 36 (30 – 47), p = 0.70), and still showed a trend toward lower RVSP with respect to the pre-surgical echo (47 (37 – 60); p = 0.10). Similar to the proportions in the first postop echo, 18 out of 32 patients (56%) demonstrated a reduction with respect to baseline, and 4 remained stable (13%). 

### Associations of baseline characteristics and volume-related parameters with change in RVSP 

**Figure 2:**
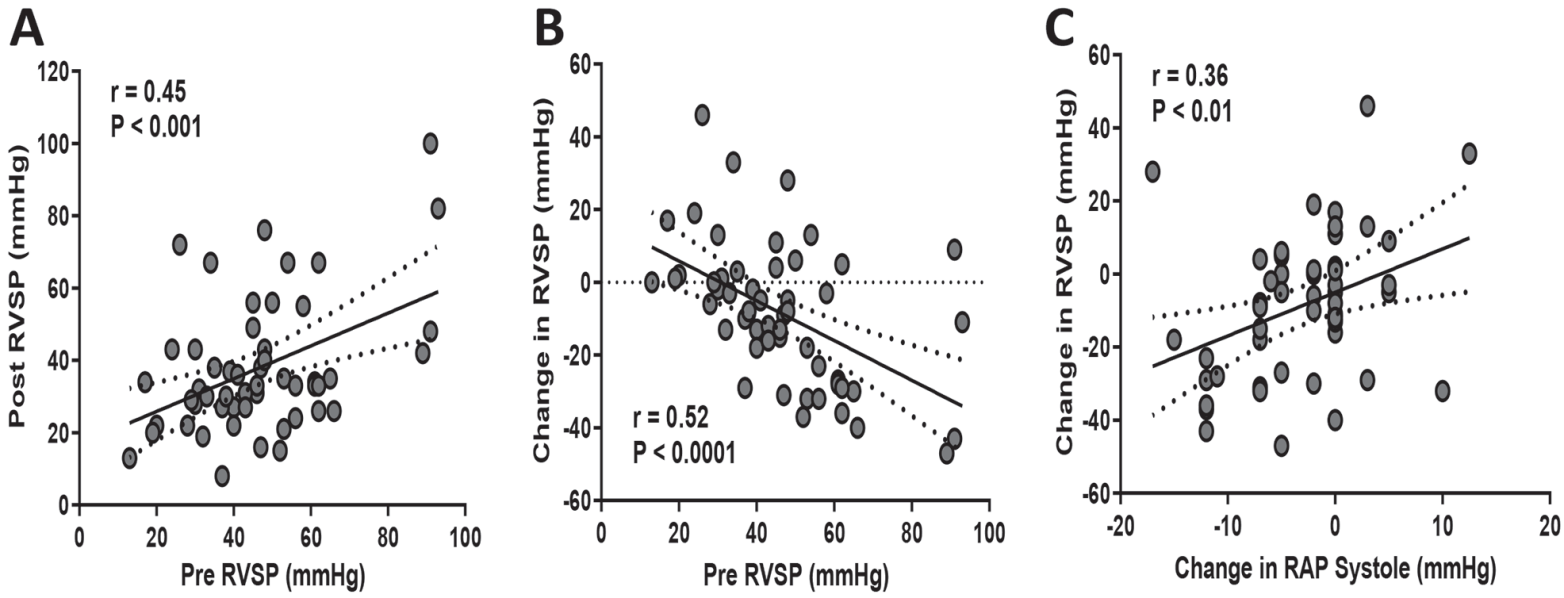
Correlations of right ventricular systolic pressure (RVSP) after arteriovenous fistula (AVF) creation. A, B: Correlation of postoperative RVSP (A) and pre-post change after AVF creation (B) with baseline RVSP measurements. C: Change in RAP during systole. Pre-post change was calculated as the postoperative value minus the pre-existing RVSP.

Postoperative RVSP significantly correlated with baseline levels, but also with the change in RVSP (calculated as postoperative value minus baseline) (Figure 2A, B). In agreement with this, a general linear regression model demonstrated that baseline RVSP (p = 0.006) was the most robust predictor of postoperative change (Table 4), with higher values prior to AVF creation associated with greater RVSP reductions after surgery. Other volume-related factors such as higher preoperative patients’ weight (p = 0.038) and the use of loop diuretics (p = 0.015) were also associated with RVSP reductions after surgery (Table 4). Pre-post change in RVSP positively correlated with change in RAP during systole, in the absence of significant changes in TR peak velocity or mitral E/E’ (Table 2) (Figure 2C), further supporting an improvement in central volume status as the main cause of postoperative RVSP reductions. No demographic characteristics or comorbidities were associated with change in RVSP (Table 3). 

## Discussion 

The AVF is the preferred vascular access for hemodialysis [40]. However, it has been traditionally implicated with cardiovascular stress and cardiac overload in ESRD patients [41, 42, 43, 44]. Several studies support the role of AVF in the development of PH [15, 18, 19, 20, 21, 22], while others suggest that access creation simply unmasks pre-existing deficiencies in cardiopulmonary circulation that are the primary culprits of PH exacerbations [15, 29, 30, 31]. The actual contribution of AVF to PH parameters is uncertain because few studies have compared pulmonary artery pressures before and after access creation [25, 28, 29, 30]. Using paired analyses of echocardiographic measurements obtained prior to AVF creation and at a median time of 1.3 years after, we demonstrate an overall improvement in RVSP values and a reduction in LVM after surgery. 

The majority of patients in our study cohort had elevated RVSP (67% with > 37 mmHg) and LVM (91% with > 122/149 g) prior to AVF creation. This is in line with previous prevalence estimates of PH and cardiac remodeling in dialysis patients [12, 14, 17, 45, 46, 47]. Cardiac remodeling in the ESRD population is the result of years with chronic volume overload, anemia, essential hypertension, and comorbid coronary or non-coronary heart disease [46, 42]. In the present study, patients with higher baseline RVSP values benefited the most from AVF creation, showing greater RVSP reductions than the rest of the patients and supporting a role for non-AVF related factors in pulmonary pressures after surgery. In a subset of patients with a second postoperative echocardiogram within 2 years of the first postop echo, post-AVF RVSP values remained stable. Altogether, these observations suggest that AVF creation by itself is not detrimental for PH. In a prospective study of 20 patients, Unal et al. [28] also reported a significant reduction in systolic pulmonary artery pressure (PAP) 2 years after AVF creation compared to baseline. 

The association of higher preoperative patients’ weight and loop diuretic use with reduction of RVSP after surgery also reflect an improvement of volume overload in these patients. Congruently, and following the same trend as RVSP, the RAP during systole demonstrated a significant reduction after AVF creation, which has shown to be an estimate of central volume status [48]. This is in line with improvements in body weight and estimated plasma volume after AVF creation and dialysis [49], and acute reductions in RVSP with hemodialysis in previous studies [50]. 

Our findings indicate that AVF can be beneficial or non-detrimental for pulmonary pressures in over 75% of the patients. In the remaining 22% who experienced RVSP increments after surgery, 4 patients showed mild to moderate increases of 4 – 9 mmHg while 8 showed increments of 11 – 46 mmHg. A leading theory about why AVF creation contributes to PH is due to increased pulmonary blood and cardiac output [18, 23, 42]. The anastomosis between the high resistance arterial system and the low resistance venous system decreases systemic vascular resistance, increases venous return, and enhances cardiac output. However, an increase in cardiac output alone does not explain PH since the pulmonary vasculature is naturally capable to adapt to variations in blood flow by vessel recruitment and dilation [15]. The echocardiographic measurement of peak early diastolic transmitral velocity to mitral annular motion velocity ratio (E/E’) in our patient population was greater than 10 in average, both pre- and post-AVF, indicating that the origin of the pulmonary hypertension in these subjects is likely to be post-capillary [51]. Upper arm AVFs have been associated with a higher risk of PH development than lower arm AVFs due to their higher blood flow and more proximal location [43], but again, none of these studies controlled for pulmonary artery pressures prior to access creation. We did not find any associations between pre-post change in RVSP and brachial vs. radial inflow of the fistula. 

The effect of AVF creation on PH remains controversial because there is no precise way to determine exactly how much a given risk factor contributes to the etiology of this disease [10, 42, 52]. The mechanism seemingly most responsible for the development of PH in CKD patients is an increase in pulmonary vascular resistance, caused by vasoconstriction and pulmonary vascular remodeling, specifically of smooth muscle cells [15]. The AVF related changes in hemodynamics superimpose non-hemodynamic factors such as uremia-induced endothelial dysfunction, vascular damage by microbubbles from the dialysis circuit, anemia, and pre-existing cardiac dysfunction [17, 47, 42]. 

Moreover, PH development or progression in dialysis patients is highly dependent on volume control and even dialyzer composition [53]. Excess volume overload or removal are associated with impaired systolic and diastolic function, especially in patients that have long interdialytic periods and high-volume accumulation [50, 54, 55]. Patients with poorly controlled volume status present with greater left and right atrial enlargement and marked elevations in RVSP in comparison with patients that have better volume control and shorter dialysis intervals [56, 57]. Patients with adequate volume control during hemodialysis show acute improvements in overall cardiac function [58]. All of these factors underscore the need for a well-controlled prospective analysis of dialysis patients to better understand the role of baseline comorbidities, access creation, and hemodialysis treatments in PH. 

The limitations of this study include the small number of patients, retrospective design, and reliance on electronic medical records for data collection. Additionally, we did not have information on AVF blood flow measurements, ultrafiltration rate, patients’ dry weight or weight changes after hemodialysis. Despite these limitations, we present pre-post data of RVSP values in patients undergoing AVF creation and demonstrate a beneficial effect of the fistula in most patients. Prospective studies in which pulmonary pressure and cardiac parameters are measured either with routine echocardiograms or invasive catheters shortly prior to AVF creation, as well as various time points after surgery and AVF usage, are needed to fully address any remaining questions and confirm these findings. Efforts to understand the factors that contribute to PH in ESRD patients are fundamental to decreasing cardiovascular mortality and improving quality of life in this population. 

## Conclusion 

Our results suggest that AVF creation and usage is associated with a significant reduction or stable measurements of RVSP in the ESRD population while reinforcing the role for non-AVF related factors in pulmonary pressures after surgery by correlating the RVSP values after AVF creation with baseline measurements. 

## Funding 

This study was supported by the National Institutes of Health grants R01-DK098511 and R01-DK121227 to RIVP, K08-HL151747 to LM, and the VA Merit Award IBX004658 to RIVP. 

## Conflict of interest 

All authors declare that they have no competing financial interests that might have influenced the present study or the preparation of the manuscript. 

**Table 1.** Baseline characteristics of the study cohort (N = 54).

Age – mean in years (± SD)	51 (± 13)
Females – N (%)	23 (43)
Ethnicity – N (%)	
African American	31 (57)
Hispanic	19 (35)
Caucasian	4 (7)
Weight – mean in kg (± SD)	78 (± 20)
BMI – mean in kg/m^2^ (± SD)	27.8 (± 6.4)
Comorbidities – N (%)	
Hypertension	53 (98)
Diabetes mellitus	37 (69)
Coronary artery disease	12 (22)
Congestive heart failure	10 (19)
*HFrEF (≤ 40%)	7 (63.6%)
*HFpEF (> 40%)	3 (36.4%)
Medications – N (%)	
ACEI/ARB	24 (45)
CCB	34 (64)
Loop diuretics	21 (40)
Beta blockers	41 (76)
Vasodilators	21 (40)
Type/location of AVF – N (%)	
Brachiobasilic	29 (54)
Brachiocephalic	15 (28)
Radiocephalic	8 (15)
Radiobasilic	1 (2)
Radioradial	1 (2)
Left upper extremity	44 (81)
History of previous AVF – N (%)	5 (9)
CKD stage 5 pre-dialysis* – N (%)	14 (26)
Baseline RVSP > 37 mmHg – N (%)	36 (67)
Baseline LVM > 122/149 g^ǂ^ – N (%)	49 (91)

*Not on hemodialysis at the time of access creation. ^ǂ^LVM > 122 for females and > 149 for males. N = number; SD = standard deviation; ACEI = angiotensin-converting enzyme inhibitors; ARB = angiotensin receptor blocker; CCB = calcium channel blockers; AVF = arteriovenous fistula; CKD = chronic kidney disease; RVSP = right ventricular systolic pressure; LVM = left ventricular mass.

**Table 2.** Echocardiographic measurements before and after AVF creation.

	Before AVF	After AVF	p
Time of echo* – median in years (IQR)	–0.3 (–0.7, –0.2)	1.3 (0.6, 2.1)	
Echo values – mean (± SD) or median (IQR)			
Tricuspid regurgitation (mmHg)	36 (27 – 42)	29 (23 – 44)	0.093
TR max velocity (m/sec)	2.77 (2.48 – 2.93)	2.52 (2.46 – 2.90)	0.88
Fractional shortening (%)	32 (± 13)	33 (± 13)	0.58
RVSP (mmHg)	46 (33 – 56)	33 (26 – 43)	0.0015
Ejection fraction (%)	55 (51 – 60)	55 (54 – 60)	0.20
Stroke volume (mL)	62 (50 – 71)	67 (59 – 74)	0.43
RAP Systole (mmHg)	10 (10 – 15)	3 (3 – 8)	< 0.001
Tricuspid E/E’ ratio (m/sec)	15 (15 – 20)	12 (10 – 15)	0.42
LVM (g)	215 (163 – 276)	201 (143 – 256)	0.045

*Time of echocardiogram with respect to AVF creation. AVF = arteriovenous fistula; IQR = interquartile range; TR = tricuspid regurgitation; RVSP = right ventricular systolic pressure; RAP = right atrial pressure; LVM = left ventricular mass. Units: mmHg = millimeters of mercury; m = meters; sec = seconds; mL = milliliters; g = grams.

**Table 3.** Associations of baseline characteristics with pre-post change in RVSP*.

	Standardized coefficient (β)	Confidence interval	p-value
Demographics and comorbidities			
Age	–0.13	–0.42, 0.16	0.38
Female sex	0.13	–0.19, 0.44	0.42
Black race	–0.10	–0.38, 0.18	0.46
Diabetes mellitus	0.22	–0.10, 0.55	0.18
Coronary artery disease	0.20	–0.08, 0.49	0.16
Congestive heart failure	–0.03	–0.32, 0.26	0.82
AVF features and history			
Brachial inflow	–0.14	–0.43, 0.15	0.34
Previous AVF	0.04	–0.26, 0.33	0.79
Pre-dialysis status	–0.02	–0.30, 0.25	0.86

*Change in RVSP was calculated as the postoperative value minus baseline. AVF = arteriovenous fistula; LVM = left ventricular mass; RVSP = right ventricular systolic pressure.

**Table 4.** Associations of factors related to volume status with pre-post change in RVSP*.

	Standardized coefficient (β)	Confidence interval	p-value
Baseline characteristics and medications			
Weight	–0.50	–0.97, –0.03	0.038
ACEI/ARB	–0.32	–0.70, 0.06	0.092
Loop diuretics	–0.59	–1.0, –0.12	0.015
Vasodilators	0.19	–0.02, 0.53	0.27
Pre-dialysis status	–0.22	–0.55, 0.11	0.18
Baseline echocardiogram parameters			
RVSP (mmHg)	–0.54	–0.93, –0.16	0.006
LVM (g)	0.13	–0.30, 0.56	0.56
Stroke volume (mL)	0.02	–0.30, 0.35	0.90
RAP systole (mmHg)	0.31	0.08, 0.70	0.12
Mitral E/E’ ratio (m/sec)	0.06	–0.34, 0.45	0.76
Pre-post change in echocardiogram parameters*			
∆ Stroke Volume (mL)	–0.23	–0.60, 0.14	0.21
∆ E/E’ (m/sec)	0.18	–0.18, 0.53	0.30

*Change in RVSP and all other echocardiographic parameters was calculated as the postoperative values minus baseline. ACEI = angiotensin converting enzyme inhibitors; ARB = angiotensin receptor blockers; RVSP = right ventricular systolic pressure; LVM = left ventricular mass; RAP = right atrial pressure; ∆ = change. Units: mmHg = millimeters of mercury; m = meters; sec = seconds; mL = milliliters; g = grams.
